# New distribution records for the critically endangered frog *Indirana gundia* (Dubois, 1986) from Kerala part of Western Ghats, India

**DOI:** 10.3897/BDJ.3.e5825

**Published:** 2015-08-11

**Authors:** Abdulrasheed Safia Jesmina, Sanil George

**Affiliations:** ‡ Rajiv Gandhi Centre for Biotechnology, Trivandrum, India

**Keywords:** Western Ghats, Distribution, *Indirana gundia*, 16S ribosomal RNA

## Abstract

**Background:**

*Indirana gundia* is one of the critically endangered frog species of Western Ghats, India, and known only from the type locality (Gundya in Karnataka State, India) at an elevation of 200 m Mean Sea Level. We provide data on the geographical distribution of this species using molecular tools.

**New information:**

Our results expand the geographical distribution range of this species about 111 km south up to the northern part of Kerala State and recorded at an elevation ranging from 115 m to 200 m asl.

## Introduction

The genus *Indirana* is the only representative of the endemic amphibian family Ranixalidae in the Western Ghats, India with twelve valid species (Modak et al. 2015). *Indirana gundia* is classified as a critically endangered frog species as per the IUCN Red List of Threatened Species (IUCN 2015) and listed as the 88^th^ of the 100 most Evolutionarily Distinct and Globally Endangered (*EDGE*) amphibians (http://www.edgeofexistence.org) of the world with an EDGE score of 5.65 (Isaac et al. 2007). The species was discovered in 1986 in the forests of Kemphole and Sakleshpur (12^o^49.50'N, 75^o^35.50'E), Karnatka, India, by Dubois 1986) and is known to occur only in the type locality (Gundya) at an altitude of 200 m asl. Morphology (Nair et al. 2012) and genetic data along with photographs (Padhye et al. 2014) of this species have been provided recently. In the absence of recent reports on the geographical distribution, the present study was designed to provide new distribution data of this species with the help of molecular tools.

## Materials and methods

**Figure 1.**
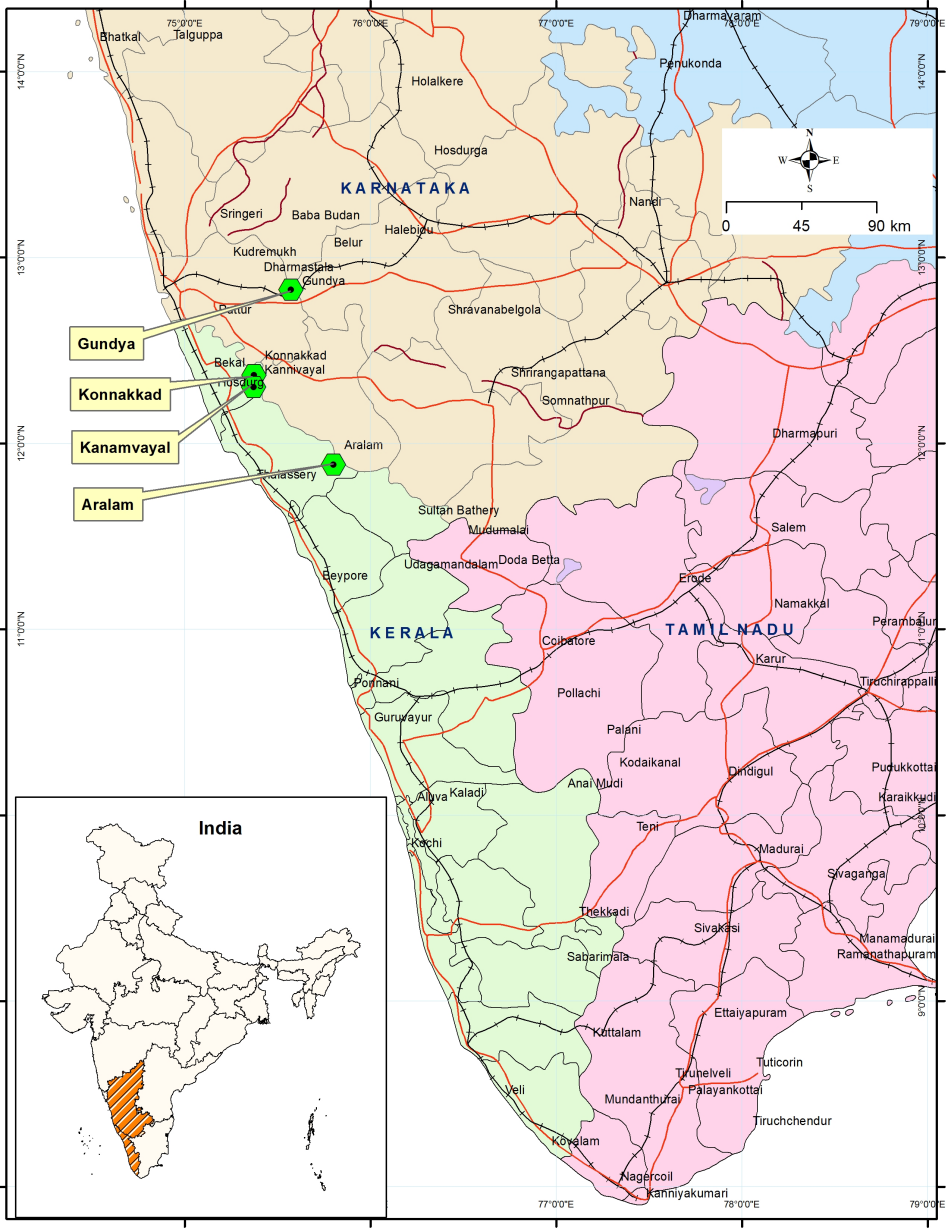
Map showing the localities where *I. gundia* occurs in Western Ghats.

**Figure 2.**
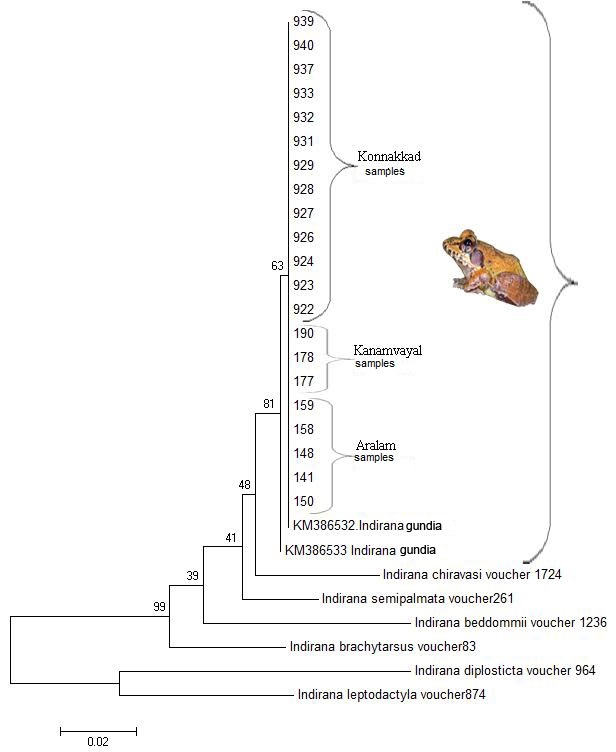
Phylogenetic tree (ML) based on 16s mitochondrial DNA sequences of *Indirana* species distributed in Kerala part of Western Ghats, India.

Visual encounter survey Method (Heyer et al. 1994) was adopted to capture thirteen individuals from Konnakkad, three from Kanamvayal and five from Aralam under the license from the Kerala Forest Department (No. WL-12-1713; Voucher numbers are given in Fig. 2). They were released immediately in their respective habitats after collecting toe clips. Since the mitochondrial DNA sequences are available for this species, we used 16S mitochondrial gene sequences to diagnose specimens from three localities: (1) Konnakkad (12°22'1"N, 75°22'21"E) (2) Kanamvayal (12°17'41.2"N, 75°28'38.0"E) and Aralam (11°52'43.7"N, 75°53'19.0"E) in the northern part of Kerala (Fig. 1). DNA was extracted from the collected toe clips using DNeasy Animal Blood and Tissue Kit (Qiagen). The genomic DNA was amplified with 16SA-L and 16SB-H primers (Palumbi et al. 1991) for 16S ribosomal RNA fragments of the mitochondrial gene as its utility in amphibian species identification is well established (Vences et al. 2005). The thermocycling conditions for the amplification of 16S gene as follows: 95°C for 5 min followed by 40 cycles of 95°C for 30s, 55°C for 40s, 72°C for 90s followed by a final extension step at 72° for 5 min. PCR products were purified and sequenced using an ABI 3730 capillary sequencer following manufacturer's instructions. We then compared the sequences with reference sequences of *I. gundia* from the type locality, available from GenBank (Acc. Numbers: KM386532 and KM386533), using BLAST. To further corroborate our results, we computed genetic distance (p-distance; Suppl. material 1) and maximum likelihood (ML) phylogenetic tree using MEGA 6 (Tamura et al. 2013) under K2+I model (Kimura 1980) as the best fitting model, along with the 16S sequences of other *Indirana* species distributed in Kerala (Fig. 2, Suppl. material 2). The obtained sequences (494 base pair in length) were deposited in GenBank under accession numbers KT282197-KT282223.

## Taxon treatments

### Indirana gundia

(Dubois, 1986)

#### Distribution

The species was discovered in 1986 from forests of Kemphole and Sakleshpur (12°49.50' N, 75°35.50' E), Karnataka, India, by Dubois and is believed to occur only in the type locality (Gundya) at an altitude of 200 m asl.

#### Taxon discussion

The GenBank sequences matched exactly the extracted sequences of *Indirana gundia* (0.1%genetic distance) and clustered together in the ML tree, strongly suggesting that the distribution range of *I. gundia* extends through the continuous stretch of forests towards south up to Aralam region of the Kerala part of Western Ghats. Our results expand the distribution of *I. gundia* about 111 km south (Aralam) of its previously known range at an elevation of 137 m mean sea level. Konnakkad is approximately 56 km south of the type locality of *I. gundia* at an elevation of 115 m mean sea level followed by Kanamvayal (61 km; 172 m). This is the first distribution record of *I. gundia* in places other than the type locality.

Geographical distribution of a species is an important parameter in conservation biology. However, the Wallacean shortfall is evident in the Western Ghats as the exact distribution of many amphibian species in this region is poorly known. The data provided here on the distributional status of one of the critically endangered frog species of Western Ghats illuminating the use of molecular tools for delineating species boundaries effectively. The results may be helpful in designing further studies on biogeography and ecology and provide valuable insights for the conservation status of this species.

## Supplementary Material

Supplementary material 1 Genetic distance (p-distance) of I. gundia samples Data type: Genetic distance
File: oo_49139.doc Abdulrasheed Safia Jesmina, Sanil George

Supplementary material 2 Aligned sequences used for the construction of ML tree Data type: DNA sequence
File: oo_49140.fas Abdulrasheed Safia Jesmina, Sanil George

XML Treatment for Indirana gundia
