# Supplementary material for: New distribution records for the critically endangered frog Indirana gundia (Dubois, 1986) from Kerala part of Western Ghats, India
Source: Biodivers Data J. 2015 Aug 11;(3):e5825. doi: 10.3897/BDJ.3.e5825 (PMC4563154; doi:10.3897/BDJ.3.e5825)
Supplement: Supplementary material 1 — Genetic distance (p-distance) of I. gundia samples [file biodiversity_data_journal-3-e5825-s001.doc]

| *I.gundia* (Type locality) |  |  |  |  |  |  |  |  |  |
| --- | --- | --- | --- | --- | --- | --- | --- | --- | --- |
| *I. gundia* (Aralam samples) | 0.0010 |  |  |  |  |  |  |  |  |
| *I. gundia* (Kanamvayal samples) | 0.0010 | 0.0000 |  |  |  |  |  |  |  |
| *I. gundia* (Konnakkad samples) | 0.0010 | 0.0000 | 0.0000 |  |  |  |  |  |  |
| *I.semipalmata* | 0.0294 | 0.0304 | 0.0304 | 0.0304 |  |  |  |  |  |
| *I.beddommii* | 0.0619 | 0.0629 | 0.0629 | 0.0629 | 0.0771 |  |  |  |  |
| *I.brachytarsus* | 0.0538 | 0.0548 | 0.0548 | 0.0548 | 0.0527 | 0.0791 |  |  |  |
| *I.chiravasi* | 0.0375 | 0.0365 | 0.0365 | 0.0365 | 0.0527 | 0.0832 | 0.0669 |  |  |
| *I.diplosticta* | 0.1389 | 0.1400 | 0.1400 | 0.1400 | 0.1339 | 0.1481 | 0.1420 | 0.1359 |  |
| *I.leptodactyla* | 0.1207 | 0.1197 | 0.1197 | 0.1197 | 0.1176 | 0.1318 | 0.1217 | 0.1237 | 0.1095 |
